# Revisiting the Role of Worries in Explaining the Link Between Test Anxiety and Test Performance

**DOI:** 10.1007/s10648-021-09601-0

**Published:** 2021-03-02

**Authors:** Frieder L. Schillinger, Jochen A. Mosbacher, Clemens Brunner, Stephan E. Vogel, Roland H. Grabner

**Affiliations:** 1 Institute of Psychology, University of Graz, Graz, Austria; 2 Department of Psychology, Technische Universität Dresden, Dresden, Germany; 3 Institute of Psychology, Ludwigsburg University of Education, Reuteallee 46, 71634 Ludwigsburg, Germany

**Keywords:** Test anxiety, Test performance, Worry, Mathematics, Education

## Abstract

The inverse relationship between test anxiety and test performance is commonly explained by test-anxious students’ tendency to worry about a test and the consequences of failing. However, other cognitive facets of test anxiety have been identified that could account for this link, including interference by test-irrelevant thoughts and lack of confidence. In this study, we compare different facets of test anxiety in predicting test performance. Seven hundred thirty university students filled out the German Test Anxiety Inventory after completing a battery of standardized tests assessing general intelligence and mathematical competencies. Multiple regressions revealed that *interference* and *lack of confidence* but not *worry* or *arousal* explained unique variance in students’ test performance. No evidence was found for a curvilinear relationship between arousal and performance. The present results call for revisiting the role of worries in explaining the test anxiety-performance link and can help educators to identify students who are especially at risk of underperforming on tests.

## Introduction

*We live in a test-conscious, test-giving culture in which the lives of people are in part determined by their test performance.*

S. B. Sarason (1959)

Tests and examinations play an important role in shaping the career of individuals, and it is therefore not surprising that students report feelings of anxiety towards tests. Numerous studies have demonstrated that test anxiety affects students in various countries and across educational levels (for reviews, see Hembree 1988; Zeidner 1998, 2007). For instance, Hill and Wigfield (1984) have estimated that about 20% of school-aged children in the USA exhibit heightened levels of test anxiety. Similar numbers have been reported for the UK (Putwain and Daly 2014) and Germany (Pixner and Kaufmann 2013). Test anxiety also remains pervasive in higher education. Work by Thomas et al. (2018) indicates that up to 25% of university students can be classified as having high levels of test anxiety, and a national survey in Germany revealed that about 13% seek counseling for test anxiety (Middendorf et al. 2016). These numbers underscore the importance of understanding the nature of test anxiety and its relationship to test performance.

### Test Anxiety and Test Performance

Since the beginning of systematic research on test anxiety in the 1950s, researchers have been interested in the question of how test anxiety affects test performance (Mandler and Sarason 1952). Cumulative evidence suggests that test anxiety is significantly and negatively related to performance in standardized tests, such as intelligence tests and aptitude/achievement tests, with small-to-medium effect sizes (for meta-analyses, see Hembree 1988; Seipp 1991; von der Embse et al. 2018). Test anxiety starts to be inversely related to test performance as early as in elementary school, and this link remains present throughout formal education (Hembree 1988; von der Embse et al. 2018). The test anxiety-performance link is also evident in the fact that test-anxious students often receive lower school and course grades than their non-anxious classmates (for meta-analyses, see Hembree 1988; Seipp 1991; von der Embse et al. 2018).

### Cognitive and Emotional Component of Test Anxiety

Given that test anxiety is linked to test performance, the question arises which specific aspects of test anxiety can explain this relationship. In fact, the phenomenology of test anxiety comprises a wide range of feelings, thoughts, and bodily responses (for a review, see Zeidner 1998). Liebert and Morris (1967) suggested differentiating between a cognitive and an emotional component of test anxiety. They identified the cognitive component as “any cognitive expression of concern about one’s own performance” (Liebert and Morris 1967, p. 975) and labeled it as *worry*. The emotional component was labeled as *emotionality*, referring to affective and autonomic reactions, such as accelerated heartbeat, sweating, or nausea.

### Differentiating Between Cognitive Facets

Whereas Liebert and Morris (1967) equated the cognitive component of test anxiety with performance-related worries, other research suggests that this component consists of different facets. Sarason (1984), for instance, identified two cognitive facets, namely *worry* (e.g., “During a difficult test, I worry whether I will pass it”) and *test-irrelevant thinking* (e.g., “During tests, I think about recent past events”). This differentiation has been further refined in the development of the German Test Anxiety Inventory (in German: Prüfungsangstfragebogen; PAF) (Hodapp et al. 1995, 2011; Hodapp and Benson 1997).

The PAF differentiates between *worry*, *interference*, and *lack of confidence* as cognitive facets and *arousal* as an emotional facet of test anxiety. *Worry* is conceptualized as in the questionnaire by Sarason (1984) (e.g., “I worry about my results”), and *arousal* refers to bodily or physical reactions in test situations (e.g., “My heart pounds”).

The interference subscale of the PAF is similar to the test-irrelevant thinking subscale by Sarason (1984) but empathizes that test-irrelevant thoughts are experienced as interfering (e.g., “I am preoccupied by other thoughts that distract me”). Cognitive interference or distraction is also assumed in prominent theoretical accounts of test anxiety (Calvo and Eysenck 1992; Eysenck et al. 2007; Wine 1971). Students are thought to shift the focus of their attention away from the task and towards inner states, which can interfere with task processing. However, these accounts identify worries as the primary source of interference/distraction and do not further differentiate between worries and test-irrelevant thoughts.

*Lack of confidence* refers to low confidence to perform well on a test (e.g., “I am convinced that I will do well”; item scores are inverted). As such, it is similar to the construct of academic self-efficacy, which describes a person’s belief that they can successfully attain educational goals (Bandura 1997; Chemers et al. 2001; Honicke and Broadbent 2016). Whereas academic self-efficacy includes beliefs regarding both test taking and study skills, *lack of confidence* refers more specifically to the confidence of a person in a test situation. Recent studies suggest that lacking confidence or control regarding a test is related to higher levels of *worry* (Cassady and Finch 2020; Putwain and Aveyard 2018; Putwain and Pescod 2018).

Having good psychometric properties (Donati et al. 2019; Hodapp et al. 2011; Hoferichter et al. 2015; Schnell et al. 2013), the PAF has become one of the most widely used questionnaires to assess test anxiety in German and has been adapted to English (Hoferichter et al. 2015) and Italian (Donati et al. 2019).

### Usage of the Term *Worry*

Differentiating between cognitive facets of test anxiety has led to ambiguity in how the term *worry* is used in the literature (see also Cassady and Johnson 2002). Whereas *worry* is sometimes used as an umbrella term to summarize cognitive as opposed to emotional aspects of test anxiety (e.g., Mowbray 2012; Seipp 1991; von der Embse et al. 2018), it is used by other researchers—and also in the present work—in a more narrow sense to refer to worries about a test and the consequences of failing (e.g., Donati et al. 2019; Hoferichter et al. 2015; Sarason 1984; Schnell et al. 2013).

### Does *Worry* Account for the Lower Test Performance?

Meta-analyses have revealed that test performance is overall more strongly related to cognitive than to emotional aspects of test anxiety (Hembree 1988; Seipp 1991; von der Embse et al. 2018). Against this background and given the particular prominence of the worry facet, worries about a test and the consequences of failing are considered by many researchers as the candidate explanation for the lower test performance of test-anxious students (e.g., Beilock et al. 2004; Brady et al. 2018; DeCaro et al. 2011; Deffenbacher 1977, 1978, 1986; Deffenbacher and Deitz 1978; Eysenck et al. 2007; Hagtvet 1976; Hembree 1988; Liebert and Morris 1967; Morris et al. 1981; Morris and Fulmer 1976; Morris and Liebert 1969, 1970; O’Neil and Fukumura 1992; Osterhouse 1972; Ramirez and Beilock 2011; Sattizahn et al. 2016; Schwarzer 1984; Steinmayr et al. 2016; Wine 1971). Worrying during a test is thought to occupy students’ minds, to disrupt effective task processing, or to consume limited working memory resources, ultimately leading to lower test performance.

The identification of different cognitive facets of test anxiety, however, raises the question of how these are differentially related to test performance. Surprisingly, the available evidence regarding this question seems to be scarce. To the best of our knowledge, only two studies have compared *worry* and *interference*/*test-irrelevant thinking* in predicting standardized test performance, and only one of these studies considered in addition *lack of confidence*. In the first study, Sarason (1984) analyzed the performance of psychology students in a digit symbol substitution test (DSST). This test involves substituting digits with symbols as fast as possible based on digit-symbol mapping. Results revealed that the performance in the DSST was lower for those students scoring high in the worry subscale but did not differ between students with low and high scores in the test-irrelevant thinking subscale. In contrast, Donati et al. (2019) found a different pattern of correlations between a standardized arithmetic test and the subscales of the PAF in Italian secondary school students. *Interference* and *lack of confidence*, but not *worry* or *arousal*, were significantly related to test performance. Similarly, mixed evidence has been reported regarding the relationship between school or course grades and different facets of test anxiety (Hodapp et al. 2011; Hoferichter et al. 2015; Putwain et al. 2020; Schnell et al. 2013).

The different tests used in the studies by Sarason (1984) and Donati et al. (2019) make it difficult to compare the results directly. Whereas the DSST is thought to assess the functioning of domain-general associative learning (for a review, see Jaeger 2018), the arithmetic test used by Donati et al. (2019) taps into domain-specific abilities, such as applying arithmetic procedures. Moreover, the results of both studies are limited by only considering bivariate correlations between test anxiety facets and performance. Bivariate correlations do not take into account shared variance between different facets of test anxiety. Given the incommensurability and limitations of previous findings, further studies are needed to determine the unique variance explained by different cognitive facets of test anxiety in test performance. Answering this question can help identify test-anxious students who are more likely to underperform on tests. This would allow educators to tailor interventions to at-risk students.

### Curvilinear Effect of Arousal

One explanation for the weaker association between performance and emotional aspects compared with cognitive aspects of test anxiety could be that intermediate levels of physical arousal help performance. According to a modern version of the Yerkes-Dodson law, there is an optimal level of arousal at which performance is maximized, with the optimum being lower for more difficult tasks (Eysenck 1982; Yerkes and Dodson 1908). Such a curvilinear relationship would not be properly reflected in linear correlations as reported by most previous studies examining how test anxiety is related to test performance (Hembree 1988; Seipp 1991; von der Embse et al. 2018). Therefore, our understanding of the test anxiety-performance link can be advanced by considering a curvilinear relationship between arousal and performance (see also Cassady and Finch 2020).

## The Present Study

The aim of the present study was to provide a fine-grained analysis of how test anxiety is related to test performance. We extend previous studies by considering different cognitive facets of test anxiety as well as a linear and a curvilinear effect of arousal in predicting performance across different standardized tests. A large sample of university students was asked to complete a battery of standardized tests assessing general intelligence and mathematical competencies, including arithmetic fact retrieval, arithmetic procedures, and higher-order mathematics. We focused on the mathematical domain since mathematics is a central part of the curriculum from an early age and as such provides an excellent touchstone for general academic performance. Moreover, mathematics has been linked in particular to performance-related anxiety as evident by a wealth of studies on the adjacent construct of math anxiety (for reviews, see Ashcraft 2002; Foley et al. 2017). Including different mathematical tests allowed us to assess the generalizability of the results within the mathematical domain and the intelligence test to probe domain-general performance. After completing the set of tests, students filled out the PAF providing three cognitive and one emotional subscale. These subscales were then used to predict performance on the standardized tests using multiple regression analyses. To ensure that findings transfer to actual academic performance, we additionally tested how the four test anxiety facets are related to the final high school grade in mathematics.

## Methods

### Participants

Data from 748 university students between the ages of 17 and 35 years were initially acquired. Seventeen data sets were excluded from the present analyses due to missing responses to one or more items of the PAF, and one data set because of an extremely low score (T-score of 1.76) in the Berlin Intelligence Structure Test (BIS-T). Thus, the final sample comprised 730 university students (470 females) with a mean age of 21.9 (*SD* = 3.3) years. There were a few cases of missing data so that sample sizes for analyses with the BIS-T (*N* = 720) and the high school grade in mathematics are lower (*N* = 710). Approximately 48% of the students were enrolled in a psychology degree, 23% in science, 18% in humanities, 6% in engineering, and 5% in law or economics. All participants gave written informed consent prior to participation and received feedback regarding their intellectual abilities after testing as incentive for taking part in the study. The study was approved by the ethics committee of the University of Graz.

### Instruments

#### German Test Anxiety Inventory

Test anxiety was assessed with the German Test Anxiety Inventory (in German: *Prüfungsangstfragebogen*, PAF; Hodapp et al. 2011). This questionnaire consists of 20 statements about feelings and thoughts in test situations. Items are rated on a 4-point Likert scale with the labels “almost never,” “sometimes,” “often,” and “almost always.” *Worry*, *interference*, *lack of confidence*, and *arousal* are differentiated with five items assigned to each subscale. *Lack of confidence* items are positively formulated and item scores were therefore inverted. Sum scores were calculated for each subscale with a minimum score of 5 and a maximum score of 20.

#### Berlin Intelligence Structure Test

The short version of the Berlin Intelligence Structure Test (BIS-T; Jäger et al. 1997) was used to assess general intelligence. This test includes 15 tasks drawing on three content components of intelligence (numerical, figural, and verbal) and four operational abilities (processing speed, memory, reasoning, and creativity). The numerical tasks required participants to continue number series (reasoning; 3 min 40 s), cross out numbers in a matrix that are larger by a factor of three as compared with the preceding number (processing speed; 1 min), memorize pairs of digits (memory; 4 min), estimate the results of complex calculation problems (reasoning; 2 min 45 s), and find different operands resulting in a given arithmetic solution (creativity; 1 min 50 s). The figural tasks required participants to remember locations on a map (memory; 3 min 10 s), complete a series of figures (reasoning; 1 min 45 s), draw emblems representing a shop (creativity; 3 min), cross out all “x” in a string of letters (processing speed; 50 s), and complete a list of drawings (reasoning; 3 min). The verbal tasks required participants to list as many abilities of a good salesperson as possible (creativity; 2 min 30 s), complete analogies (reasoning; 1 min 30 s), rate statements whether they are an opinion or a fact (reasoning; 1 min), answer short questions to a memorized text (memory; 3 min), and cross out words that are meronyms of the preceding word (e.g., “word” preceded by “letter”; processing speed; 40 s). Tasks were presented in mixed order, and the test lasted for approximately 47 min including a warm-up task and instructions. Scores were aggregated across the three subscales yielding a general intelligence score.

#### Arithmetic Fluency Test

The Arithmetic Fluency Test (AF-T) is designed to measure the ease or fluency with which individuals can solve multiplications, additions, and subtractions (Schillinger et al. 2018; Vogel et al. 2017, 2019). Arithmetic problems are grouped by operation on separate sheets, and participants have to solve as many problems as possible on each sheet within a limited time. The *arithmetic fact subtest* includes 64 single-digit multiplications (e.g., 5 × 7), 128 single-digit additions (e.g., 4 + 7), and 128 subtractions with a minuend between 4 and 20 and a single-digit subtrahend (e.g., 16 − 8). For each of the three sheets/operations, participants are given 90 s. Adults have been repeatedly shown to solve such simple arithmetic problems by retrieving the respective solution from long-term memory (e.g., Grabner and De Smedt 2011). The *arithmetic procedure subtest* includes 60 problems for each operation, and participants are given 120 s per sheet/operation. The multiplications are composed of a double-digit number (smaller than 100) and a single-digit number (e.g., 39 × 5), additions require to sum up three double-digit numbers (e.g., 30 + 98 + 59), and subtractions consist of two double-digit numbers (e.g., 82 − 31). Such complex arithmetic problems usually require the application of arithmetic procedures to be solved (e.g., Grabner and De Smedt 2011). For each subtest, a total score was obtained by counting the number of correctly solved items.

#### Mathematics Test for Selection of Personnel

Performance in higher-order mathematics was assessed with the short version of the German Mathematics Test for Selection of Personnel (*Mathematiktest für die Personalauswahl*, M-PA; Jasper and Wagener 2011). The M-PA was developed to assess mathematical competencies of applicants with at least a lower secondary education degree between the ages of 16 and 40 years. The short version consists of 31 mathematical problems with a multiple-choice (MC) or open-answer (OA) format. Problems cover a wide range of mathematical topics including fractions (3 OA), conversion of units (3 OA), exponentiation (7 OA), division with decimals (2 OA), algebra (1 MC), geometry (1 MC), roots (7 OA), and logarithms (7 OA). Following instructions, participants had a total of 15 min to solve the problems. The short version of the M-PA has been reported to have good internal consistency (Cronbach’s alpha = .89) and to be highly correlated with the long version of the M-PA (*r* = .93) (Jasper and Wagener 2011). The total number of correctly solved items was used in the analysis.

### Procedure

Data collection took place between 2015 and 2019. The test battery was advertised as an assessment of personality traits and intellectual abilities, and volunteers were tested in small groups from four to twelve persons. Assessing intellectual abilities in a group setting is known to be ego-involving, and the present test situation is therefore likely to evoke performance pressure similar to actual academic tests (DeCaro et al. 2011; Mandler and Sarason 1952; Nguyen and Ryan 2008; S. B. Sarason and Mandler 1952). In addition, all standardized tests were timed, which has been suggested to be a major stressor for test-anxious students with detrimental effects on test performance (Faust et al. 1996; Morris and Liebert 1969; Onwuegbuzie and Seaman 1995).

Upon arriving, participants were seated in front of the test booklet, which included all tests and questionnaires described in the “Instruments” section. In addition, tests assessing creativity and personality as well as questionnaires on math anxiety and general anxiety were administered. These additional instruments are not within the scope of the present study and are therefore not reported. The sequence of tests was as follows: BIS-T, M-PA, AF-T, creativity test, personality test, PAF, math anxiety questionnaires, and general anxiety questionnaire. Participants were instructed to work through the booklet page by page and to pause whenever they reached a page with a red stop sign. For all standardized tests, experimenters took the time to inform participants when they had to stop working on the respective test. At the end of the test booklet, participants were asked to fill out demographic questions regarding sex, age, field of study, and final high school grade in mathematics. Testing took about two and a half hours and was conducted by one of four trained student research assistants.

### Data Analysis

Analyses were run in the R environment (R Core Team 2015) with specific packages listed in the following description. All data, scripts, and results are available at the open science framework (OSF): https://osf.io/tszeg.

To facilitate a direct comparison between performance measures, mean scores of the BIS-T, AF-T, and M-PA were transformed to a common T-scale (*M* = 50, *SD* = 10). Grades in mathematics were inverted so that higher values represent better grades (5 = very good, 4 = good, 3 = sufficient, 2 = poor, 1 = insufficient).

As a first step, we tested whether the four PAF subscales reflect distinct facets of test anxiety by computing the internal consistency (Cronbach’s alpha) of each subscale as well as the correlations between subscales. We then inspected the bivariate correlations between test anxiety facets and performance measures, and finally used multiple linear regressions to determine the unique variance that test anxiety facets explained in students’ test performance. The assumptions for the multiple linear models were checked by inspecting diagnostic plots in combination with running the following tests: heteroscedasticity was tested by studentized Breusch-Pagan tests (Breusch and Pagan 1979; Koenker 1981), multicollinearity was checked by computing the variance inflation factor (VIF), and influential observations were defined based on Cook’s distance. Self-reported grades in mathematics were predicted with an ordinal regression model, checking the assumption of proportional odds with a likelihood ratio test (“ordinal” package; Christensen 2019). Note that only three students reported having received the lowest grade in mathematics, resulting in a disproportional low number of observations in this particular cell. We therefore excluded these data points for analyses involving grades. Finally, a curvilinear relationship between *arousal* and performance was examined for each linear model by testing whether the model fit improved significantly by including a quadratic term of the predictor *arousal*.

## Results

### Scale Characteristics

**Fig. 1:**
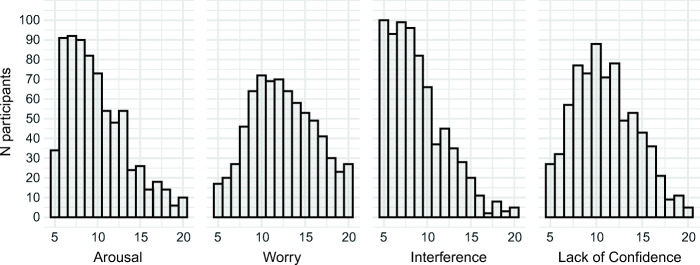
Distribution of test anxiety facets. Histograms show the distribution of the arousal, worry, interference, and lack of confidence subscale of the German Test Anxiety Inventory (PAF) with sum scores on the abscissa and number of participants on the ordinate

Distributions of the four PAF subscales are depicted in Fig. 1. The internal consistency was good for all subscales (arousal: *α* = .85; worry: *α* = .81; interference: *α* = .84; lack of confidence: *α* = .87). At the same time, the correlations between the three cognitive subscales were low-to-medium (see Table 1) confirming that *worry*, *interference,* and *lack of confidence* are distinct cognitive facets of test anxiety.

**Table 1:** Bivariate correlations between variables used in the multiple regression analyses

	Test anxiety				Test performance				
	Arousal	Worry	Interference	LOC	Intelligence	Facts	Procedures	Mathematics	Grade
Arousal	—								
Worry	.34***	—							
Interference	.36***	.21***	—						
LOC	.52***	.25***	.48***	—					
Intelligence	−.11**	−.09*	−.16***	−.15***	—				
Facts	−.11**	−.03	−.19***	−.21***	.53***	—			
Procedures	−.05	−.01	−.18***	−.17***	.47***	.77***	—		
Mathematics	−.07	−.08*	−.14***	−.14***	.39***	.37***	.38***	—	
Grade	.01	-.01	−.20***	−.15***	.28***	.22***	.26***	.38***	—

Spearman’s rank correlation coefficient is reported for all correlations with the grade in mathematics. Pearson’s correlation coefficient was computed for all other correlations

*LOC* lack of confidence

*p < .05; **p < .01; ***p < .001

### Bivariate Correlations

**Fig. 2:**
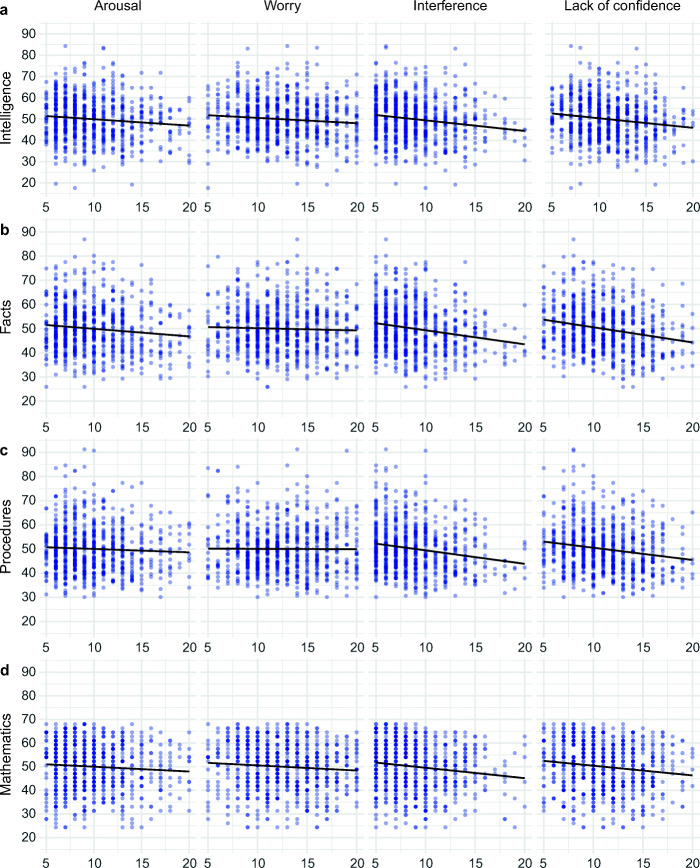
Test performance as a function of test anxiety facets. Scatter plots depict sample-based t-scores of tests assessing **a** general intelligence, **b** arithmetic fact retrieval, **c** arithmetic procedures, and **d** higher-order mathematics on the ordinate across panels. Sum scores of the test anxiety subscales are depicted on the abscissa across panels. Darker blue represents overlap of data points

Bivariate correlations (see Table 1) showed that *arousal* was inversely related to intelligence and arithmetic fact retrieval but not to arithmetic procedures or higher-order mathematics. *Worry* was significantly correlated to intelligence and higher-order mathematics but not to arithmetic fact retrieval or arithmetic procedures. In contrast, *interference* and *lack of confidence* showed highly significant negative correlations with all performance measures. Test performance as a function of the four test anxiety facets is depicted in Fig. 2.

**Fig. 3:**
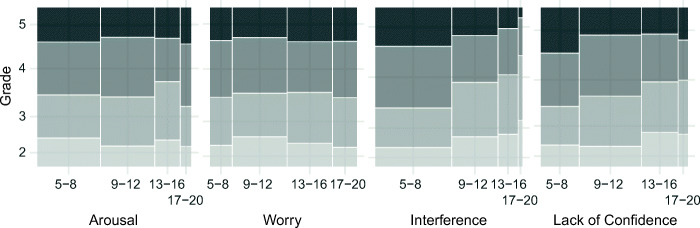
Mathematics grade as a function of test anxiety facets. Mosaic plots depict the final high school grade in mathematics on the ordinate across panels. Sum scores of the test anxiety subscales are depicted on the abscissa. For illustrative purposes, each subscale is divided into four equidistant segments. The width of the grayed rectangles represents the proportion of sum scores falling into a segment, whereas the height represents the proportion of grades within a segment. Higher values represent better grades

A similar pattern of results was found regarding the grade in mathematics. Whereas *interference* and *lack of confidence* were negatively correlated with grade, *worry* and *arousal* did not show a significant relationship. Mathematics grade as a function of the four test anxiety facets is depicted in Fig. 3.

### Multiple Linear Regressions

Diagnostic plots indicated that the distribution of the residuals deviated from normality for predicting arithmetic procedures and higher-order mathematics. Given the comparatively large sample size of the present study, this deviation was deemed acceptable (see Schmidt and Finan 2018). Homoscedasticity was given for all models as confirmed by Breusch-Pagan tests (lowest *p* value = .30), and multicollinearity was low (highest VIF = 1.58). Cook’s distance indicated that there were a few cases of influential observations in each model. We therefore used robust linear regression (“robust base” package; Maechler et al. 2009) to ensure that results did not depend on influential observations.

Predictor estimates and overall model fit for the four linear regression models are summarized in Table 2. In line with the literature, *arousal* was not significantly related to test performance. Surprisingly, *worry* did not remain significantly related to any performance measure. *Interference* and *lack of confidence*, instead, explained unique variance in performance across tests. Taken together, test anxiety accounted for 2 to 5% of the variance in students’ test performance.

**Table 2:** Estimates and overall model fit of test anxiety facets predicting test performance

	Intelligence				Facts				Procedures				Mathematics			
	*E*	*SE*	*t*	*p*	*E*	*SE*	*t*	*p*	*E*	*SE*	*t*	*p*	*E*	*SE*	*t*	*p*
(Intercept)	57.46	1.57	36.58	< .001	56.62	1.59	35.50	< .001	53.32	1.59	33.41	< .001	57.35	1.75	32.65	< .001
Arousal	−0.02	0.12	−0.17	.862	0.01	0.12	0.14	.886	0.18	0.12	1.55	.121	0.07	0.14	0.54	.583
Worry	−0.11	0.10	−1.11	.264	0.07	0.10	0.73	.461	0.15	0.11	1.37	.169	−0.14	0.11	−1.34	.180
Interference	−0.34	0.12	−2.77	.005	−0.34	0.12	−2.85	.004	−0.40	0.12	−3.29	.001	−0.29	0.13	−2.11	.035
LOC	−0.25	0.13	−1.93	.053	−0.46	0.13	−3.54	<.001	−0.39	0.12	−3.07	.002	−0.30	0.14	−2.02	.043
Explained variance	*R*^2^_adj_ = .033				*R*^2^_adj_ = .046				*R*^2^_adj_ = .040				*R*^2^_adj_ = .024			
Model fit	*χ*^2^(4) = 28.11, *p* < .001				*χ*^2^(4) = 39.26, *p* < .001				*χ*^2^(4) = 32.11, *p* < .001				*χ*^2^(4) = 21.61, *p* < .001			

*LOC* lack of confidence, *E* estimate, *SE* standard error

Ordinal regression was used to test how the different facets of test anxiety relate to the grade in mathematics. Likelihood ratio tests confirmed that the assumption of proportional odds was given for all predictors.

Predictor estimates and overall model fit for the ordinal regression model are summarized in Table 3. Similar to standardized test performance, *interference* and *lack of confidence* remained inversely related to the final high school grade in mathematics. In addition, *arousal* was found to exhibit a positive relationship. Students reporting higher arousal in test situations received a better grade in mathematics than students reporting lower arousal when controlling for the other facets of test anxiety. Again, *worry* did not explain unique variance in students’ mathematics grade. Taken together, test anxiety accounted for approximately 2% of the variance in the final high school grade in mathematics.

**Table 3:** Estimates and overall model fit of test anxiety facets predicting grade in mathematics

	*E*	*SE*	*z*	*p*
Arousal	0.06	0.02	2.92	.003
Worry	0.01	0.01	0.61	.538
Interference	−0.11	0.02	−4.60	< .001
LOC	−0.07	0.02	−2.71	.006
Explained variance	*R*^2^_McFadden_ = 0.02			
Model fit	*χ*^2^(4) = 42.74, *p* < .001			

*LOC* lack of confidence, *E* estimate, *SE* standard error

Table 4 summarizes the results of testing for a curvilinear relationship between *arousal* and performance. The model fit did not improve significantly by including a quadratic term of the predictor *arousal* for any of the above-described models (see also Figs. 2 and 3). Further information regarding the specifications of all tested models is available at the OSF repository.

**Table 4:** Testing for a curvilinear relationship between arousal and performance

DV	*χ*^2^	*df χ*^2^	*p*
Intelligence	0.04	1	.834
Facts	2.07	1	.150
Procedures	0.07	1	.788
Mathematics	0.17	1	.684
Grade	0.41	1	.523

Comparison of the model fit between a model with four linear predictors (i.e., arousal, worry, interference, lack of confidence) and a model which includes an additional quadric term for the predictor *arousal*

*DV* dependent variable

## Discussion

### Cognitive Facets and Test Performance

We first addressed the question of how the different cognitive facets of test anxiety are related to test performance. Previous studies have provided mixed evidence regarding this question but were limited by only considering bivariate correlations between test anxiety facets and performance indictors (Donati et al. 2019; Hodapp et al. 2011; Hoferichter et al. 2015; Sarason 1984; Schnell et al. 2013). We could overcome this limitation in the present study by using multiple regression analyses to predict test performance in a large sample of university students. Moreover, the battery of tests used in the present study allowed us to test the generalizability of the results within the mathematical domain and to probe domain-general performance.

On a bivariate level, *worry* was found to be significantly related to general intelligence and higher-order mathematics (see Table 1 and Fig. 2). However, correlation coefficients were small and there was no significant correlation between *worry* and arithmetic nor between *worry* and final high school grade in mathematics (see Fig. 3). In contrast, *interference* and *lack of confidence* were consistently related to lower performance across standardized tests as well as lower grades in mathematics. Multiple regressions revealed that *worry* did not explain unique variance in students’ test performance. In contrast, *interference* and *lack of confidence* remained significantly related to standardized test performance and mathematics grade.

The present results replicate the correlations between PAF subscales and arithmetic performance reported by Donati et al. (2019). In addition, we show that *interference* and *lack of confidence* explain unique variance in both arithmetic fact retrieval and arithmetic procedures. Importantly, the same pattern of results was found in the domain-general intelligence test. This is in contrast with findings by Sarason (1984) according to which associative learning is inversely related to *worry* but not to *test-irrelevant thinking*. One explanation for this discrepancy could be the different conceptualization of the interference/test-irrelevant thinking subscale. Whereas the scale reported by Sarason (1984) is assessing test-irrelevant thoughts in general, the interference subscale used in the present study as well as by Donati et al. (2019) refers more specifically to test-irrelevant thoughts that are experienced as distracting or interfering.

### Arousal and Test Performance

Arousal was significantly and negatively correlated with general intelligence and arithmetic fact retrieval. However, multiple regressions revealed that arousal did not explain unique variance in standardized test performance. Controlling for the other facets of test anxiety, arousal turned out to be positively related to the final high school grade in mathematics.

Including a quadratic term of the predictor *arousal* did not improve the model fit for any performance measure. The present study therefore does not provide evidence for a curvilinear relationship between arousal and performance as predicted by the Yerkes-Dodson law (Eysenck 1985; Yerkes and Dodson 1908).

### Revisiting the Role of Worries

Performance-related worries are considered by many authors as the candidate explanation for the lower test performance of test-anxious students (e.g., Beilock et al. 2004; Brady et al. 2018; DeCaro et al. 2011; Deffenbacher 1977, 1978, 1986; Deffenbacher and Deitz 1978; Eysenck et al. 2007; Hagtvet 1976; Hembree 1988; Liebert and Morris 1967; Morris et al. 1981; Morris and Fulmer 1976; Morris and Liebert 1969, 1970; O’Neil and Fukumura 1992; Osterhouse 1972; Ramirez and Beilock 2011; Sattizahn et al. 2016; Schwarzer 1984; Steinmayr et al. 2016; Wine 1971). The present results, in contrast, suggest that worries cannot explain the lower test performance of test-anxious students. Instead, interference by test-irrelevant thoughts and lack of confidence seem to be the driving forces behind the inverse relationship between test anxiety and test performance. This is at odds with experimental studies showing adverse effects of worries on working memory processes (for reviews, see Eysenck et al. 2007; Moran 2016).

Hayes et al. (2008), for instance, asked lower and higher test-anxious students to either think about a personal topic that had been bothering them (worry condition) or about a positive topic (positive thought condition) while performing a random generation task. In a random generation task, participants are asked to produce a random sequence of items (e.g., by pressing different buttons) avoiding repeating or stereotyped patterns—a process with relatively high working memory demands. Comparing the randomness of the produced sequences between groups (lower vs. higher test anxiety) and conditions (worry vs. positive thought) revealed that there was no difference between groups in the positive thought condition. In the worry condition, however, higher test-anxious students showed an inferior performance as compared with lower test-anxious students. These results suggest that worries can have a detrimental effect on test-anxious students’ working memory.

Given the correlational nature of the present study, we cannot rule out that worrisome thoughts did affect the working memory of students which would eventually impair their test performance. However, the detrimental effect of worries seems to be negligible compared with the effects of being distracted by test-irrelevant thoughts and lacking confidence. The present results therefore suggest revisiting the role of worries in explaining the link between test anxiety and test performance.

### Psychological Mechanisms

What are the psychological mechanisms that could explain the present findings? *Interference* and *lack of confidence* are similar to *worry* given that they are cognitive instead of emotional in nature (see Liebert and Morris 1967). Yet, previous research has demonstrated that *worry*, *interference*, and *lack of confidence* are distinct facets of test anxiety, which is corroborated by the present findings (Donati et al. 2019; Hodapp et al. 2011; Hoferichter et al. 2015; Schnell et al. 2013).

In contrast to worries, test-irrelevant thoughts are not focused on performance or possible consequences of failing. Instead, students report seemingly random thoughts that pop into their minds (Hodapp et al. 2011; Sarason 1984; Zeidner 1998). For instance, a student might wonder whether they locked their apartment in the middle of taking a test. Such thoughts are likely to impair students’ abilities to solve the task at hand by dividing their attention between test-relevant and test-irrelevant thoughts. Impaired attention has also been suggested to underlie the detrimental effect of worries on test performance (for a review, see Wine 1971). Here, however, the extra burden would be put on students’ minds by unrelated thoughts instead of test-related worries. To maintain test performance, test-anxious students would need to inhibit these thoughts and to re-direct their attention to the task at hand. Such inhibiting processes have been identified as a basic function of the central executive (Miyake et al. 2000), which are known to vary between individuals (for a review see Miyake and Friedman 2012). Executive functions are therefore likely to moderate the relationship between *interference* and test performance (see also O’Donnell 2017).

Lacking confidence, on the other hand, could diminish the perseverance of test-anxious students during a test. They might just give up working on difficult problems more quickly than less test-anxious students. Spending less time on the task would directly result in a lower test performance. At the same time, students with skill or test-taking deficits are more likely to develop lower levels of confidence regarding tests (for a review, see Tobias 1985). This could lead to a vicious cycle in which lower test performance will further decrease the confidence of test-anxious students. A lower task engagement could also leave students more room to think about unrelated topics during a test, in turn, increasing interference by test-irrelevant thoughts.

### Limitations and Directions for Future Research

In the present study, we focused on the domain of mathematics as a touchstone of academic performance. This raises the question of whether the present results also apply in a general way to other academic domains, such as reading and writing. The fact that we found the same pattern of results between test anxiety and a domain-general intelligence test, including several verbal items, as between test anxiety and mathematical performance would suggest so. However, further studies are needed to test the relationship between different test anxiety facets and performance in other academic domains more systematically.

Another limitation concerns the generalizability of the results beyond university students. In general, previous studies have reported similar results regarding the test anxiety-performance link for different age groups (for meta-analyses, see Hembree 1988; Seipp 1991; von der Embse et al. 2018). Importantly, Donati et al. (2019) have shown that *interference* and *lack of confidence* but not *worry* are inversely related to mathematical test performance in secondary school students. However, to the best of our knowledge, no study has yet addressed the role of different facets of test anxiety in explaining test performance in primary school students. This age group would be especially important to investigate since it is known that the relationship between test anxiety and test performance starts to emerge during this time (for a review, see Hill and Wigfield 1984).

Finally, the present study cannot resolve the question of causality between *interference* or *lack of confidence* and test performance given its correlational nature. To answer this question, intervention or experimental studies would be needed. For instance, future studies could aim to reduce the degree to which students experience interference by test-irrelevant thoughts. Comparing students’ test performance before and after the intervention would allow for assessing the causal effect that *interference* has on performance.

## Conclusions

Our results suggest that the inverse relationship between test anxiety and test performance is related to interference by test-irrelevant thoughts and lack of confidence. This challenges the widely accepted notion according to which the test performance of test-anxious students is impaired by worries about a test and the consequences of failing. No evidence was found for a linear or curvilinear relationship between arousal and test performance. Future research should address the role of different test anxiety facets in other academic domains than mathematics as well as in primary school students. Moreover, experimental or intervention studies are needed to pinpoint the causal effect of *interference* and *lack of confidence* on students’ test performance. The present results can help educators to identify students who are especially at risk of underperforming in tests.

## Data Availability

All data and results are available at the Open Science Framework (https://osf.io/tszeg).

## Code Availability

R code used for the analyses is available at the Open Science Framework (https://osf.io/tszeg).
